# PET-Based Imaging with ^18^F-FDG and ^18^F-NaF to Assess Inflammation and Microcalcification in Atherosclerosis and Other Vascular and Thrombotic Disorders

**DOI:** 10.3390/diagnostics11122234

**Published:** 2021-11-29

**Authors:** William Y. Raynor, Peter Sang Uk Park, Austin J. Borja, Yusha Sun, Thomas J. Werner, Sze Jia Ng, Hui Chong Lau, Poul Flemming Høilund-Carlsen, Abass Alavi, Mona-Elisabeth Revheim

**Affiliations:** 1 Department of Radiology, Hospital of the University of Pennsylvania, 3400 Spruce Street, Philadelphia, PA 19104, USA; william.raynor@pennmedicine.upenn.edu (W.Y.R.); peter.park@pennmedicine.upenn.edu (P.S.U.P.); austin.borja@pennmedicine.upenn.edu (A.J.B.); tom.werner@pennmedicine.upenn.edu (T.J.W.); abass.alavi@pennmedicine.upenn.edu (A.A.); 2 Perelman School of Medicine at the University of Pennsylvania, 3400 Civic Center Boulevard, Philadelphia, PA 19104, USA; yusha.sun@pennmedicine.upenn.edu; 3 Department of Medicine, Crozer-Chester Medical Center, Upland, PA 19013, USA; szejia.ng@crozer.org (S.J.N.); hui.chong.lau@crozer.org (H.C.L.); 4 Department of Nuclear Medicine, Odense University Hospital, 5000 Odense C, Denmark; pfhc@rsyd.dk; 5 Department of Clinical Research, University of Southern Denmark, 5000 Odense C, Denmark; 6 Division of Radiology and Nuclear Medicine, Oslo University Hospital, Sognsvannsveien 20, 0372 Oslo, Norway; 7 Institute of Clinical Medicine, Faculty of Medicine, University of Oslo, Problemveien 7, 0315 Oslo, Norway

**Keywords:** atherosclerosis, ^18^F-sodium fluoride, NaF, ^18^F-fluorodeoxyglucose, FDG, PET, calcification, vasculitis, thrombosis, IgG4-RD

## Abstract

Positron emission tomography (PET) imaging with ^18^F-fluorodeoxyglucose (FDG) represents a method of detecting and characterizing arterial wall inflammation, with potential applications in the early assessment of vascular disorders such as atherosclerosis. By portraying early-stage molecular changes, FDG-PET findings have previously been shown to correlate with atherosclerosis progression. In addition, recent studies have suggested that microcalcification revealed by ^18^F-sodium fluoride (NaF) may be more sensitive at detecting atherogenic changes compared to FDG-PET. In this review, we summarize the roles of FDG and NaF in the assessment of atherosclerosis and discuss the role of global assessment in quantification of the vascular disease burden. Furthermore, we will review the emerging applications of FDG-PET in various vascular disorders, including pulmonary embolism, as well as inflammatory and infectious vascular diseases.

## 1. Introduction

Atherosclerosis is the leading cause of cardiovascular diseases (CVDs) [1]. Globally, 31% of all deaths in 2016 were caused by CVD, of which 85% were due to heart attacks or strokes [2]. Atherosclerotic changes of the vasculature can be detected through different imaging techniques and can be divided into two main categories centering either on the degree of stenosis or on plaque composition. Previously, the clinical focus has been on measuring the degree of the stenosis and the subsequent physiological effect. To assess the cardiac physiology, radionuclide ventriculography, often referred to as a MUGA (multiple-gated acquisition) scan, where a gamma camera following an injection of radioactively labeled red blood cells is used to measure the left ventricular ejection fraction (LVEF). Echocardiography is another technique that uses sound waves to produce images of the heart and, also, allows for the assessment of LVEF. Myocardial perfusion scintigraphy has traditionally used single-photon emission computed tomography (SPECT), but more recently, positron emission tomography (PET) is also increasingly used in the diagnosis of ischemic chest pain and for the evaluation of known coronary artery disease (CAD).

In contrast to these techniques, imaging modalities able to characterize plaque causing lumen stenosis have been the main focus the last decade due to increased knowledge of the pathogenesis of the atherosclerotic process. At present, it is well-known that narrowing of the arterial lumen by atherosclerotic plaques or macrocalcifications is incapable of predicting plaque rupture and, consequently, thrombosis in the affected vessels [3,4,5]. Computed tomography (CT) permits the detection and characterization of macrocalcification in atherosclerosis [6]. Cardiac CT has been demonstrated to have utility in assessing early atherosclerotic changes, including signs of inflammation, in addition to plaque burden and degree of plaque calcification [7,8,9]. The macroscopic calcification evident on CT may, however, not be a reliable predictor for future cardiovascular events. Heavily calcified plaques are associated with a more stable disease and are less vulnerable to rupture and, consequently, thrombosis in the affected vessels [10,11]. Atherosclerosis is a chronic, systemic disease with inflammation as the dynamic trigger for progression [1,12,13]. Vulnerable or high-risk plaques are characterized by a necrotic core, the infiltration of macrophages, increased network of vasa vasorum, and microcalcification [11,14]. Since plaques can rupture without any preceding clinical symptoms, changes in their morphology over time are difficult to predict; evaluating patients’ overall vulnerability rather than the individual plaques could be of greater value for risk assessments and treatment decisions [15,16]. Thus, imaging techniques that can evaluate cellular processes preceding plaque rupture and allow for a global assessment of the disease burden for the patient at the early stages will be critical for the prevention of further disease progression and subsequent clinical complications. ^18^F-fluorodeoxyglucose (FDG) is taken up by metabolically active cells in the plaques conjectured to be inflammatory macrophages [3,17,18], while ^18^F-sodium fluoride (NaF) is deposited at the sites of microcalcification due to physicochemical exchange of the ^18^F^-^ ion with the hydroxyl group in hydroxyapatite [19,20,21,22]. Hence, fused PET/CT with FDG and NaF can visualize atherosclerotic disease on a molecular scale earlier in the disease progression when changes may still be reversible (Table 1 and Table 2).

## 2. Atherosclerosis

### 2.1. Role of FDG in Atherosclerosis

Atherosclerosis, or atherosclerotic cardiovascular disease, is a chronic condition characterized by arterial stiffening due to the buildup of cholesterol plaques on vessel walls [65]. Endothelial cell dysfunction is believed to underlie the pathogenesis of atherosclerosis. In brief, hypertension and hyperlipidemia contribute to the upregulation of endothelial cell adhesion molecules [66]. The resultant recruitment of inflammatory cells propagates the inflammatory cascade, including platelet activation, deposition of lipid plaques, smooth muscle proliferation, and, ultimately, vessel micro- and macrocalcifications [13]. Progressive enlargement of these plaques throughout the body leads to a spectrum of debilitating cardiovascular conditions, including peripheral artery disease, ischemic stroke, coronary artery disease, and acute myocardial infarctions [67]. These conditions represent a major cause of morbidity and mortality both in the Unites States and worldwide [68,69,70]. Therefore, effective strategies to identify atherosclerotic disease early in the disease pathogenesis, as well as to quantify the extent of disease burden, are alluring.

**Figure 1:**
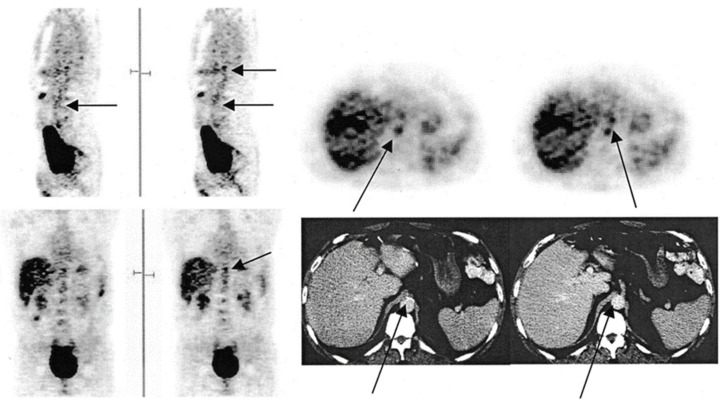
FDG-PET images illustrating the foci of FDG uptake along the aorta. In the sagittal view, the arrow in the left image indicates the abdominal aorta, while the top arrow on the right points to the budding superior mesenteric artery. In the transverse view, the bottom and top arrows indicate the abdominal aorta and budding superior mesenteric artery, respectively. In the coronal view, the arrow points to the budding mesenteric artery. In the CT image, the left arrow points to calcification along the abdominal aorta, while the right indicates to the budding superior mesenteric artery (from Yun et al. [23] with permission).

Conventional imaging modalities, including ultrasonography, CT, and MRI angiography, are widely used clinically to visualize large symptomatic plaques but are limited in their ability to assess the early stages of atherosclerosis [71,72]. In contrast, molecular imaging offers a tantalizing opportunity to examine the pathological hallmarks of atherosclerotic disease at the microscopic level [73]. As discussed previously, FDG demonstrates remarkable sensitivity and specificity for inflammatory lesions (Figure 1). Further, FDG was postulated to be effective in the identification of the inflammatory precursor lesions that precede calcific atherosclerotic disease. Yun et al. first examined vascular FDG uptake in 137 patients who underwent FDG-PET scanning [23]. They observed that over half of the subjects demonstrated vascular FDG uptake, with a greater prevalence among older individuals. Further studies demonstrated that vascular inflammation as assessed by FDG was associated with proinflammatory molecular and cellular markers of atherosclerosis [24,25,26].

It is becoming apparent, however, that the association between FDG uptake and risk factors associated with disease progression may be less straightforward than originally postulated. Yun et al. demonstrated in a later study of 156 patients that intravascular FDG uptake was significantly related to age and high cholesterol but not other cardiovascular risk factors, including cigarette use, diabetes mellitus, hypertension, and obesity [27]. Other researchers have demonstrated similar results using a variety of protocols and parameters to further characterize the clinical impact of arterial FDG uptake [3,28,29,30,40,41,42,74,75]. While the results of these studies are challenging to compare directly, in general, FDG uptake demonstrates a clear association with age but only a vague relationship with other risk factors [31,32]. For example, Pasha et al. measured the tissue-to-background ratio and weighted-average mean standardized uptake value to examine 76 patients who underwent FDG-PET/CT imaging and found that patients with cardiovascular risk factors had increased FDG uptake in the aorta but not in the peripheral (i.e., femoral and iliac) arteries [30]. Due to this variability, careful interpretation and clinical correlation should be applied to focal vascular FDG uptake.

Moreover, FDG has been found to demonstrate a low specificity for the future development of calcifications [33,34,35,36,76]. The uptake of FDG by endothelial cells and smooth muscle cells increases in hyperinflammatory states such as cancer, thereby potentially obfuscating the localization and quantification of FDG uptake due to atherogenic activity [5]. Meanwhile, stable disease, which may present with substantial plaque burdens but minimal or variable inflammation, similarly obscure the FDG-PET findings, what Meirelles et al. described as the “waxing and waning” effect [37]. That is, while focal FDG uptake is frequently observed in atherosclerotic disease, it has not been clearly associated with the structural manifestations of atherosclerosis identifiable by CT. Interestingly, arterial macrocalcification detected by CT has been shown to regress in angina patients over a 2-year period, suggesting that structural changes associated with atherosclerosis may also not be stable for measurements over time [43]. While further longitudinal studies should be performed to corroborate the results, the variability of FDG uptake during atherosclerosis progression further challenges the temporal use of FDG-PET/CT.

Numerous studies have investigated the relationship between FDG uptake within vulnerable plaques and the risk for future cardiovascular outcomes [31,77,78]. For example, the FDG uptake at plaques found in carotid arteries was found to be higher in patients who experienced early recurrent strokes [79]. An association between high FDG uptake and plaques with high-risk morphological features has been confirmed histologically as well [80]. Despite the apparent positive results, however, there remains significant challenges to using FDG-PET to study atherosclerotic plaques. First are the technical challenges intrinsic to FDG-PET, such as low specificity and resolution; the accurate measurement of FDG uptake in plaques can be hampered by high physiological myocardial FDG uptake, small diameter of the arteries, and cardiac motion [5,81]. A decreasing myocardial FDG uptake requires prior adherence to a high-fat, low-carbohydrate diet, which can be hard to follow for patients [82,83]. Furthermore, the absence of FDG uptake in the plaque may not always indicate a truly negative result, as it could be due to the insensitivity of PET to detect small foci of the FDG uptake [84].

Another argument against the use of FDG-PET for studying atherosclerotic plaques is the limited clinical significance of the vulnerable plaque; it is well-known that plaques can rupture without any preceding or warning symptoms, the morphology of plaques detected by imaging modalities can vary over periods of time, and plaque lesions that rupture are often previously characterized as non-culprits. Furthermore, only a small number of vulnerable plaque ruptures cause actual symptomatic events. Therefore, FDG-PET should not be limited in its scope to examining specific plaques. Rather, it should be used for deriving atherosclerotic burdens measured from FDG uptakes in broader anatomical structures and vessels [74].

The last general limitation of FDG that must be mentioned is its inability to elucidate the precise cellular mechanism of disease progression and its relationship with organs of high intrinsic glucose uptake. Despite this, FDG-PET has been used to rationalize the mechanistic relationship between CVD and neuropsychiatric conditions through bone marrow and spleen involvement [85,86]. For instance, it has been found that a high FDG uptake in the amygdala correlates with CVD events, arterial inflammation, and FDG uptake in the bone marrow and spleen, which was taken to rationalize that stress may lead to CVD events through the increased production of inflammatory cells from the hematopoietic stem cell niche [38]. However, the main metabolic activity of the bone marrow that accounts for a high FDG uptake is the production of red blood cells, which varies widely among subjects of different ages, rather than the generation of inflammatory cells [87,88,89]. Similarly, FDG uptake in the spleen is known to differ based on the clinical context [90]. Therefore, only relying on FDG-PET to draw specific cellular mechanisms and causal relationships between CVD and organs of high natural FDG uptake should be avoided.

In the current state of research, the prognostic value and implementation of FDG-PET for the assessment of the atherosclerotic risk remain to be further tested. There has been no clear association between the FDG uptake and CT calcium burden, nor a prospective study in noncancerous patients that correlates an increased FDG uptake with adverse cardiovascular outcomes [91]. The CAMONA (Cardio-vascular Molecular Calcification Assessed by ^18^F-NaF PET/CT) study involving 50 patients with angina pectoris and 89 healthy controls, for instance, revealed no significant correlation of FDG uptake in the thoracic aorta and 10-year Framingham Risk Score (FRS) [34]. Additionally, the variability in the protocols and reporting outcomes further confounded the implementation of FDG-PET. A review of 49 articles using FDG-PET to evaluate atherosclerosis inflammation revealed 53 different acquisition protocols and 46 methods of quantify the tracer uptake. Standardization and harmonization of the method, therefore, remains an essential step to be taken before using FDG-PET in clinical practice [92].

### 2.2. Role of NaF in Atherosclerosis

Over the past decade, increasing attention has turned toward NaF-PET/CT to detect vascular microcalcifications. Unlike FDG, NaF is not taken up by metabolically active tissues such as the myocardium, which allows NaF-PET to have a greater sensitivity and less background uptake than FDG for the assessment of CVDs [76]. In their methodological piece, Irkle et al. demonstrated that NaF has demonstrated a sensitivity for calcification in the vascular tissue, thereby lending itself well to atherosclerotic disease [93]. Research using vascular NaF-PET/CT has demonstrated that coronary; pulmonary; and peripheral (i.e., aorta, carotid, iliac, and femoral) artery NaF uptake is significantly correlated with a number of cardiovascular risk factors, including age, BMI, diabetes, hypertension, hyperlipidemia, and cardiovascular events; however, it is not associated with smoking and is variably associated with sex [41,44,45,46].

**Figure 2:**
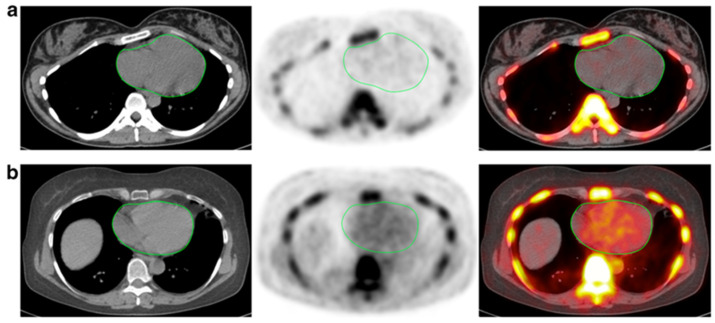
CT, NaF-PET, and fused NaF-PET/CT images of clinically normal (**a**) 25- and (**b**) 61-year -old subjects’ hearts. Green line delineates the region of interest around the heart analyzed to calculate the global cardiac calcification scores, which are 12,492.44 (**a**) and 18,424.70 (**b**). Despite the relatively increased NaF uptake in the PET scan of the subject’s heart (**b**), there is no visible calcification in the corresponding CT scan. The disparity between two modalities alludes to CT-visible macrocalcification as end-stage disease process, while NaF uptake may reflect early pathological, molecular changes (from Raynor et al. [94] with permission).

Evidence points toward NaF uptake as a significant clinical metric for atherosclerosis (Figure 2). Kwiecinski at al. and Kitagawa et al. both found that focal coronary NaF uptake on the index scans significantly correlates with the incidence of myocardial infarction [47,48]. As such, findings on NaF-PET/CT may therefore serve as a tool to assess the future risk of atherosclerosis complications. Other studies by Rojulpote et al. and Patil et al. have correlated the NaF uptake of vital and laboratory values such as blood pressure and the triglycerides-to-high-density lipoprotein ratio, respectively [49,50]. In addition, NaF uptake has been associated with widely used clinical scores for cardiovascular disease burden, including the Framingham Risk Score, atherosclerotic cardiovascular disease (ASCVD) risk scores calculated by the Pooled Cohort Equation, and CHADS_2_/CHADS_2_-VASc [34,51,52,53,54].

**Figure 3:**
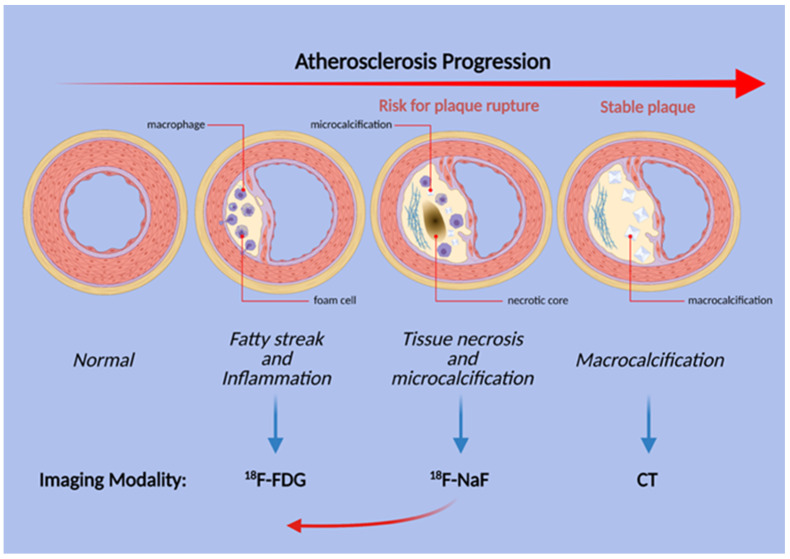
Schematic illustration of the stages of atherosclerosis in the coronary arteries. Uptake of both FDG and NaF is evident before the structural changes are visible, but inflammation and FDG uptake does not necessarily precede microcalcification. Thus, NaF uptake may be present earlier than previously thought (red arrow).

It is clear that molecular imaging will play a major role in atherosclerotic imaging in future clinical practice (Figure 3). Recent evidence has pointed toward NaF as a more useful clinical tool than FDG in the evaluation of atherosclerotic disease, particularly for a high plaque burden. In an examination of 19 multiple myeloma patients who underwent NaF- and FDG-PET/CT scans, Li et al. found that increased NaF uptake was associated with increased plaque density, while the inverse was true for FDG [55]. With regard to the pathological development of atherosclerotic plaques, a number of studies have observed that vascular NaF uptake is greater in high-risk lesions than stable plaques in the carotid and coronary arteries [14,56,57]. In addition, Ishiwata et al. recorded the initial arterial NaF uptake in the abdominal aorta and common iliac arteries and then tracked the atherosclerotic disease progression using CT. They observed that, on index scans, NaF uptake was greater in noncalcified than calcified lesions, perhaps reflecting active plaque deposition; however, the initial NaF findings did not correlate with the disease burden determined by CT alone during follow-up at 1 to 2 years [55,58].

Regardless, there exists a paucity of longitudinal, prospective research utilizing repeat NaF-PET scanning to examine the development and progression of NaF-avid lesions and vessel wall calcifications [95]. There currently has not been any human studies establishing a clear link between arterial NaF uptake by macrocalcification and the subsequent transformation into CT-detectable macrocalcification; the clearest association with early NaF uptake and corresponding coronary macrocalcification was demonstrated using an Ossabaw miniature swine model for metabolic syndrome [19]. Therefore, the development of well-powered, prognostic studies conducted with longitudinal design remains necessary to fill this void and confirm the potential of NaF-PET for the clinical assessment of atherosclerosis [91].

Another parameter that should be examined with caution in PET research design is the use of the target-to-blood pool ratio (TBR), which is derived by dividing the raw standard uptake value (SUV) to the venous blood pool SUV [25]. This derivation attempts to calibrate for the assumed background tracer activity in the blood but currently remains a controversial and even unreliable method. For instance, no clear biological rationale is offered for its use. Furthermore, TBR calculations can introduce large variability to the data, since the venous blood pool SUV is often minimal and affected by wide-ranging factors such as the venous blood flow rate, blood cell uptake, and individual differences in FDG clearance [96]. Blomberg et al. highlighted the unreliability of the TBR method when the authors found that the TBR values calculated at 1, 2, and 3 h after tracer administration were inconsistent [39].

### 2.3. Alavi-Carlsen Calcification Score (ACCS)

In lieu of the TBR method, global assessment of the major vessels, called the Alavi-Carlsen Calcification Score (ACCS), may offer significant advantages for using PET imaging to study diffuse CVD activity [97]. The Alavi-Carlsen Calcification Score method of global assessment contrasts with the focal approach, which is limited to specific sites such as atherosclerotic plaques in the coronary arteries [81]. A limitation of measuring the focal uptake in such small vessels arises from the insufficient resolution of most PET scanners, which has the potential to underestimate the associated radiotracer uptake [59,98]. Evaluating the major vessels could overcome this limitation, considering that calcification in the thoracic aorta is shown to strongly correlate with the coronary artery score [99,100].

As such, the ACCS global assessment examines atherosclerosis in its appropriate context as a diffuse, systemic disease that exerts differential effects in various parts of the affected arteries [97,101]. The score is derived from measuring the total tracer uptake in structures such as the entire body, major vessels, or specific organs such as the heart in the form of average SUV over a broad segment of the body. It allows for the measurement of the atherosclerotic burden in the early stages of the disease progression, unlike the method of measuring plaque uptakes that occur in later phases. The regions of interest (ROIs) can easily be defined based on gross structures seen in CT or the cardiac silhouette in 3D. Since the score is measured based on clearly defined and delineated anatomical boundaries, it is less subject to human bias and variations in measurements, even allowing for artificial intelligence (AI)-based approaches with a reproducibility of 100% [91].

The ACCS global assessment approach has been employed to demonstrate that patients with multiple myeloma have a higher uptake of NaF in the thoracic aorta and whole heart, as measured by the target-to-background ratio (TBR) compared to a matched control group [60]. Similarly, the CVD risk factors, such as total cholesterol in patients with type 2 diabetes mellitus, have been shown to be associated with increased NaF uptake when measured as the global TBR in the femoral arteries [61]. A retrospective analysis of 86 healthy controls and 50 patients with persistent chest pain revealed using the ACCS approach of NaF uptake in the whole heart as measured by the mean standardized uptake value (SUVmean) was higher in patients compared to the control subjects and could be employed to retrospectively predict the patient status [62]. Overall, these studies demonstrate the suitability and potential of quantifying disease risk through global assessment, of which the latter has now become an attractive option that is quick and easy to perform, especially using artificial intelligence-based processing [63,64,97,101].

### 2.4. Other PET Tracers in Atherosclerosis

The most used radiotracer in PET imaging is FDG, which has well-studied roles in atherosclerosis imaging. However, FDG has several limitations. FDG accumulates in all cells that metabolize glucose, and a high physiologic myocardial uptake obscures the uptake due to the presence of macrophages in atherosclerotic plaques. ^68^Ga-DOTATATE is a tracer that was originally intended for the improved detection of somatostatin receptor 2 (SSRT2)-positive neuroendocrine tumors. SSTR2 is also expressed on plaque macrophages; hence, it has the potential to visualize vulnerable plaques. Tarkin et al. [102] tested the efficacy of ^68^Ga-DOTATATE compared to FDG in 42 patients with atherosclerosis, and ^68^Ga-DOTATATE was shown to differentiate culprit lesions from non-culprit lesions better than images obtained by FDG. In addition, its degree of uptake correlates with the Framingham cardiovascular risk score.

Translocator protein (TSPO) ligands expressed on the macrophage in the process of plaque formation can be targeted by the C-PK11195 tracer. In an animal model, Laitinen et al. [103] demonstrated that tracer uptake was higher in inflamed than in noninflamed plaques but that other healthy structures of the artery wall also had prominent uptake, limiting its potential utility.

The expression of C-X-C motif chemokine receptor 4 (CXCR4) and its endogenous ligands and C-X-C motif chemokine 12 (CXCL12) can be found in cardiac myocytes and fibroblasts [104]. According to Hu et al., there is an upregulation of CXCR4/CXCL12 in response to hypoxia in myocardial infarction, which, in turn, initiates the process of recruitment of cardioprotective cells to protect the myocardium from reperfusion damage [105]. Recently, several studies have demonstrated promising results of the possibility of using CXCR4-directed ^68^Ga-Pentixafor PET/CT imaging to evaluate atherosclerotic plaque lesions [106,107,108]. These prompt further studies to compare CXCR4-directed ^68^Ga-Pentixafor PET/CT imaging with imaging using FDG. Kircher et al. conducted a retrospective study to compare the performance between CXCR4-directed ^68^Ga-Pentixafor PET/CT and FDG-PET/CT in detecting the atherosclerotic lesion, showing that the former was able to visualize more plaque lesions than the latter. Apart from macrophages, CXCR4 could be expressed in thrombocytes, T cells, and smooth muscle cells, representing ^68^Ga-Pentixafor might be able to detect early-stage lesions without the setting of marked inflammation [109]. As discussed in an earlier section, FDG uptake is only associated significantly with age. A study conducted by Weiberg et al. successfully established that CXCR4-directed ^68^Ga-Pentixafor uptake has a marked association with different cardiovascular risk factors, including age, arterial hypertension, and history of smoking [110]. The ability of ^68^Ga-Pentixafor to detect lesions earlier and its association with cardiovascular risk factors make it a promising alternative for FDG imaging.

## 3. Thrombotic Disorders

### 3.1. FDG-PET in Pulmonary Embolism

**Figure 4:**
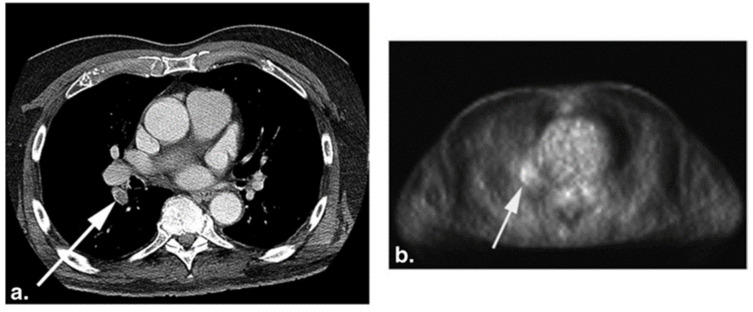
CT (**a**) and the corresponding FDG-PET (**b**) images of a 75-year-old woman with a history of melanoma. The white arrows point to pulmonary embolism (PE) present in the right lower lobe segmental artery. Increased FDG uptake (**b**) is seen at the location of PE on the PET image (from Flavell et al. [119] with permission).

Pulmonary embolism (PE) is the obstruction of the pulmonary artery and/or its branches by occlusive materials such as thrombus or tumor samples that could lead to sudden death by infarction if left untreated. The clinical symptoms of PE such as dyspnea and chest pain are often non-sensitive and nonspecific, making objective testing such as the D-dimer test or CT pulmonary angiography crucial for its diagnosis and subsequential therapy [111]. While noninvasive imaging modalities such as CT angiography remain as the gold standard, the evaluation PE is unlikely to be replaced by FDG-PET; there have been reports of FDG-PET in allowing clinicians to identify PE in the setting of oncological practices where FDG is routinely used (Figure 4). In fact, there have been numerous case reports of incidentally detecting PE as lesions with a focal FDG uptake using FDG-PET/CT [112,113,114,115,116]. Although the exact physiological mechanism remains unclear, it is hypothesized that the increased presence of inflammatory cells such as neutrophils at the site of embolism is responsible for the FDG uptake [117,118].

A retrospective study of thirteen patients with coincidental acute PE who underwent FDG-PET as a part of oncological treatment revealed that regions of acute pulmonary embolism have greater FDG activity compared to vessels without thrombi, with the shapes of the uptakes being focal or curvilinear. Specifically, the mean SUV of acute PE was 1.65 ± 0.61, while vessels with no thrombus was 1.15 ± 0.38, with a *p*-value of 0.009. An increased FDG uptake represented as a focal or curvilinear abnormality in the PET scans corresponded to the contrast-enhanced CT abnormality identified as PE [117]. Another retrospective study performed by Flavell et al. examining 59 FDG-PET/CT studies of patients with incidental PE similarly demonstrated that there was an increased focal FDG uptake within the pulmonary artery involved in or next to the PE compared to normal arteries. Furthermore, three of the cases with identified pulmonary infarct exhibited associated FDG avidity. Interestingly, one of the studies revealed that increased FDG uptake in the right ventricular wall was associated with saddle PE and bowing of the interventricular septum [119].

The current literature supports that FDG-PET may be used to differentiate a pulmonary embolism from other diseases of the pulmonary artery, such as pulmonary artery sarcoma. Pulmonary artery sarcoma is a rare malignancy rising from the mesenchymal cells of the pulmonary artery, often mimicking PE in clinical presentation and imaging [120]. A study of three patients with pulmonary artery sarcoma and ten patients with proximal PE demonstrated that the mean SUVmax of pulmonary artery sarcoma lesions (7.63 ± 2.21) was significantly higher than that of PE (2.31 ± 0.41), with a *p*-value of 0.011 [118]. A retrospective study by Lee et al. with eighteen subjects similarly found that the SUVmax of the malignant pulmonary artery lesions (10.2 ± 10.8) was significantly higher than that associated with PE (1.7 ± 0.3, *p* < 0.001) [121]. A literature review determined that a FDG SUVmax cutoff value of 3.3 had the sensitivity, specificity, and accuracy of 98.4%, 96.8%, and 97.8%, respectively, for discriminating malignant pulmonary artery lesions [122]. Regardless, further studies on the use of FDG-PET with a larger study cohort remains necessary to fully understand and establish its potential for detecting and diagnosing PE.

### 3.2. FDG-PET in Deep Vein Thrombosis and Venous Thromboembolism

Deep vein thrombosis (DVT) is the formation of thrombus in a deep vein, such as the femoral vein; prompt diagnosis and treatment are crucial to avoid venous thromboembolism (VTE), which is a deadly complication of DVT followed by PE. The risk factors for thrombosis are delineated by Virchow’s triad, which refers to a state of hypercoagulability, endothelial vessel injury, and venous stasis [123]. Ultrasonography (US) is the primary method of detecting DVT; incompressibility of a venous vessel with a US probe is considered diagnostic of DVT [124]. While US have many advantages, such as the lack of radiation exposure and accessibility, they heavily rely on interpreter and operators’ experiences, and obtaining quality US images can be stymied by the patient’s state of health, such as obesity and edema [125].

Several articles have reported the use and advantage of FDG-PET for the identification of DVT. Hara et al. demonstrated using a murine stasis-induced DVT model with which FDG-PET/CT could identify DVT by detecting neutrophil-dependent inflammation of the thrombus. The same study also retrospectively examined 19 DVT patients and found that FDG uptake in the vein with DVT as measured by SUVmax and TBR was significantly greater compared to that of the matched control patients and that the FDG signal within DVT decreased over time [126]. In another study of twelve symptomatic patients with DVT by Rondina et al., the SUVmax values of the thrombosed vein regions were found to be significantly greater than that of the contralateral leg without thrombosis. The SUVmax threshold of greater than or equal to 1.645 was 87.5% sensitive and 100% specific for DVT. Furthermore, the SUVmax of the thrombosed vein regions decreased over time, suggesting that the metabolic activity of a vein with thrombosis can be quantitatively correlated to the time since the onset of DVT symptoms [125]. Hess et al. showed that, in fifteen patients with suspected DVT and/or PE, FDG-PET/CT accurately detected DVT in all patients, while the results for PE were unclear, with only two out of six patients demonstrating FDG avidity [127]. Zhu et al. found an association between lower leg venous FDG uptake and the risk of developing VTE in a retrospective study with 10 patients [128].

In contrast to the positive studies, Le Roux et al. concluded that FDG-PET/CT may not be accurate enough for the diagnosis of VTE. This study of 100 patients found low sensitivities of FDG-PET/CT for both PE and VTE (3% and 31%, respectively). Although FDG uptakes in regions with DVT were significantly higher compared to the corresponding contralateral vessels, the authors could not find any SUVmax threshold significant to being used as a diagnostic cutoff [129]. Additionally of interest, studies employing FDG-PET/CT to distinguish venous thrombosis from other types of thrombosis, such as those of tumor or septic origin, reported no significant FDG uptakes in simple venous thrombosis [130,131]. Further studies investigating and differentiating the use of FDG-PET in a wide range of thrombi with the appropriate time points and controls could help clarify and consolidate the results of the various studies.

Overall, the advantages of FDG-PET in detecting DVT include the examination of anatomical locations not easily accessible by US, comprehensive whole-body image acquisition, and the quantification of FDG uptake to deduce the timeline of the thrombus [132]. While it is unlikely that FDG-PET will replace US in diagnosing DVT, FDG-PET may serve key roles in detecting PE or DVT in oncological patients who often undergo FDG-PET imaging as a part of their routine care. Cancer patients have a higher risk of developing DVT, and a retrospective investigation of 131 cancer patients with a history of DVT or PE, who underwent FDG-PET/CT imaging, revealed abnormal FDG venous uptakes in 26 (19.8%) patients, with the most common site of thrombosis being the inferior vena cava [133]. Employing FDG-PET/CT to detect DVT early in oncology patients may significantly improve the therapeutic outcomes and significantly decrease the comorbidities of cancer.

## 4. FDG beyond CVDs

### 4.1. FDG-PET in Large Vessel Vasculitis

Large vessel vasculitis (LVV), which includes Takayasu arteritis (TA) and giant cell arteritis (GCA), affects large vessels such as the aorta and its branches, causing chronic granulomatous inflammation. TA commonly affects women between the ages of 15 and 30 years, while GCA is more common after 50 years of age [134]. Although temporal artery biopsy is the gold standard for the diagnosis of GCA, imaging has played an increasing role in the diagnosis of both types of LVV [135]. Modalities such as ultrasounds, CT, magnetic resonance imaging (MRI), and PET/CT have been proposed to assist in the evaluation of vasculitis. Compared to anatomic modalities, PET/CT has the advantage of portraying molecular changes before morphological ones become manifest. Increased glycolytic activity by the macrophages and lymphocytes present in the arterial wall in the LVV results in an increased FDG uptake [136]. Unlike the patchy FDG uptake resulting from atherosclerosis, LVV causes a mural FDG uptake that is smooth and circumferential [137].

**Figure 5:**
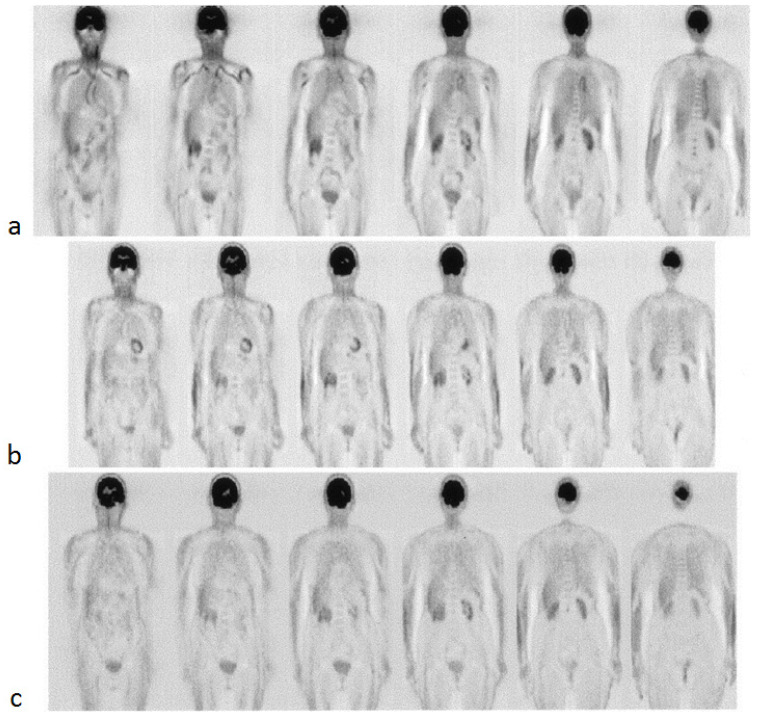
FDG-PET images of a 78-year-old woman with giant cell arteritis at the baseline (**a**), 3 months of therapy (**b**), and 6 months of therapy (**c**). High-tracer uptake is initially present and visible in the thoracic aorta and subclavian arteries, as evident in the first two images from the left (**a**), which progressively decreased after treatment with steroids (from Blockmans et al. [142] with permission).

Several recent studies have proposed that FDG-PET/CT has a role in the diagnosis and monitoring of GCA [137,138,139,140,141]. In this disease, the symmetric involvement of the subclavian arteries and aorta often demonstrate increased FDG activity (Figure 5) [142]. A study by Sammel et al. considered 64 cases of suspected GCA and compared the imaging obtained within 72 h of starting glucocorticoids with the results of the temporal artery biopsy [139]. The sensitivity of FDG-PET/CT in the diagnosis of GCA was determined to be 92%, while the specificity was 85%. The importance of FDG-PET/CT in instances of suspected GCA with a negative temporal artery biopsy was evaluated in a study by Hay et al., who showed a large vessel uptake of FDG in 22 out of 63 such cases [140]. FDG-PET/CT has also been found to have a potential role in the diagnosis of TA, in which involvement of the left subclavian artery and bilateral carotid arteries is more common [135,143]. A prospective evaluation of 30 TA patients found that FDG could portray local inflammation and vascular remodeling [144]. A meta-analysis that included 191 TA patients across seven studies determined that FDG-PET had a pooled sensitivity of 87% and a pooled specificity of 73% [145].

Polymyalgia rheumatica (PMR) is a rare disease found exclusively in adults over the age of 50 and characterized clinically by morning stiffness and aching at the shoulders, hip girdle, torso, and neck. It is a disease that involves the proximal articular and periarticular structures (bursae and tendons) and can be associated with giant cell arteritis (GCA). The occurrence of PMR is approximately 50% in patients with GCA [146]. Henckaerts et al. examined the FDG uptake in 99 patients who underwent FDG-PET scanning. They observed 67% sensitivity and 87.5% specificity by using FDG-PET in diagnosing the affected patients with a high clinical suspicion of PMR, and the diagnostic accuracy improves before commencement of the glucocorticoid treatment [147]. FDG-PET studies have revealed a characteristic FDG uptake by the bursitis in particular joints, mainly glenohumeral; sternoclavicular; spinous processes; greater trochanters; and, to a lesser extent, in the wrists, elbows, and acromioclavicular joints [148]. A meta-analysis of 636 patients across nine studies found that FDG uptake at certain joints such as hips, ischial tuberosities, shoulders, and sternoclavicular yield a higher positive likelihood ratio of PMR [149].

In addition to the diagnosis of LVV, FDG-PET/CT may have a role in prognostication and evaluating the response to treatment. Glucocorticoids are often the first-line treatment for LVV, along with methotrexate, cyclophosphamide, and tocilizumab also available as therapeutic options. A retrospective study of 12 GCA patients treated with glucocorticoid and tocilizumab revealed that complete remission in all subjects was accompanied by a significant decrease in the FDG SUVmean [150]. In a study of 56 LVV patients, Grayson et al. observed that a higher vascular FDG uptake in patients in clinical remission was associated with eventual clinical relapse [151]. Similarly, a higher total lesion glycolysis was detected in patients with complicated progress compared to patients with favorable progress in 17 cases of confirmed LVV [152]. Muratore et al. followed 93 patients with LVV for a median of 31 months and discovered that the baseline FDG uptake was associated with a greater risk of aortic dilatation [153]. Based on these results, PET/CT has a promising role in the diagnosis and monitoring of LVV disease activity over time, with the potential to assess the outcomes and influence the management. Due to the systemic nature of vasculitis, total body imaging may offer greater sensitivity and specificity for these pathologies [154]. As sensitive imaging modalities play a growing role in assessing LVV, longitudinal prospective studies are necessary to validate and standardize assessments with FDG-PET/CT.

### 4.2. FDG-PET in Vascular Diseases of Infectious Etiology

Although less common, other disorders affecting the cardiovascular system may benefit from the unique perspective offered by PET/CT imaging. As a sensitive indicator of inflammation and infection, FDG may play a role in the diagnosis and monitoring of infectious aortitis [155,156,157]. Murakami et al. found that the maximum SUV (SUVmax) was higher in all cases of infected aortic aneurysms compared to noninfected cases in a total of 11 patients [157]. Additionally, FDG uptake has been observed to normalize after the successful treatment of infectious aortitis before the response was visible on CT [156]. Other types of infections, such as vascular prosthetic graft infection (VPGI), can also be visualized by FDG-PET/CT. A meta-analysis of 286 cases of VPGI across 10 studies found that FDG-PET or FDG-PET/CT had a pooled sensitivity of 96% and a pooled specificity of 74% [158]. Incidentally, intramural hematomas, aortic dissection, arterial pseudoaneurysms, and endoleaks after endovascular aneurysm repair can be visualized by FDG-PET/CT [137]. These rare conditions and incidental findings should be taken into consideration in the interpretation of PET/CT images, and suspected vascular infection may benefit from imaging with FDG-PET/CT in cases that are difficult to diagnose.

One of the minor criteria in the Duke criteria for infective endocarditis is a mycotic aneurysm, which is a relatively rare infected aortic aneurysm with an extremely high mortality rate; therefore, it is crucial to diagnose it early. As described earlier, increased FDG uptake is noted in inflammatory lesions. This suggests that FDG-PET might have an advantage in diagnosing mycotic aneurysms. Murakami et al. demonstrated the possibility of utilizing FDG-PET/CT in the diagnosis of mycotic aneurysms [157]. A few case reports have shown that the sensitivity of FDG-PET/CT in detecting mycotic aneurysms is higher compared to transesophageal echocardiography and CT [159,160,161]. Apart from diagnosing mycotic aneurysms, FDG-PET/CT could be used to assess the effectiveness of antibiotic therapy. In the study of Morimoto et al., there was a decreased FDG uptake in follow-up FDG PET/CT scans of patients who received antibiotics therapy [161].

### 4.3. FDG-PET in Vascular Diseases of Immunoglobulin G4-Related Disease (IgG4-RD)

Immunoglobulin G4-related disease (IgG4-RD) is an immune-mediated fibroinflammatory disease that can involve multiple organ systems [162,163]. The disease is characterized by IgG4-expressing plasma cell organ infiltration, obliterative phlebitis, and storiform fibrosis. IgG4-RD may affect the vascular system of large-to-medium-sized vessel walls and coronary arteries [164,165,166]. IgG4-related aortitis and/or periaortitis are commonly located in the infrarenal abdominal aorta and iliac arteries [164]. The disease is usually responsive to immunosuppressants but can have severe effects if left untreated, and early recognition of the disease is crucial [167]. Diffuse circumferential thickening and homogenous increased FDG uptake of the walls with or without aortic dilatation is typically seen on FDG-PET/CT. There may sometimes also be focal stenosis with hypermetabolic pseudotumor [168,169]. In a study by Zhang et al., all patients diagnosed with IgG4-RD were found to have hypermetabolic lesion(s) on FDG-PET/CT, and 97.1% (34/35) of these patients showed multiorgan involvement. As many as 25/35 (71.4%) patients had more organ involvement detected by FDG-PET/CT than by conventional evaluations, including a physical examination, ultrasonography, and CT [169]. Another study by Huang et al. with 12 patients assessed the utility of FDG-PET/CT in guiding biopsies for difficult sites, such as the coronary arteries, for the diagnoses of IgG4-RD, in addition to evaluating the disease response and recurrence [166]. Several studies reported an emerging role of FDG-PET/CT for assessing organ involvement, monitoring the therapeutic response, and guiding the interventional treatment of IgG4-RD [169,170,171].

## 5. Conclusions

The role of PET in vascular disorders continues to grow as its utility in diagnosis and disease monitoring is validated in a variety of inflammatory and infectious conditions. Vascular inflammation as portrayed by FDG, which may play a limited role in assessing atherosclerosis, can also be used in diverse vascular diseases, including thrombotic disorders and vasculitis. NaF, on the other hand, has emerged as a highly sensitive and specific marker of vascular microcalcification in both the major vessels, such as the aorta, and in the coronary arteries. NaF-PET/CT stands promising for the early global assessment of atherosclerosis; however, future prospective and longitudinal studies designed to establish a clear link between NaF uptake and visible macrocalcification remain warranted. Finally, continuous investigation of the use and mechanism of FDG-PET in vascular disorders has the potential to not only complement traditional imaging modalities but also enhance patientcare by allowing prompt diagnosis and treatment.

## Figures and Tables

**Table 1:** Studies on the Role of FDG in atherosclerosis.

Author Publication Year Country [Ref. #]	Subjects	Objectives	*n* (Female) Age in Years, Mean ± SD or Range	Arterial Segments	Findings
Yun et al. April 2001. PA, USA. [23]	Patients who have undergone FDG-PET scans.	To evaluate the vascular uptake in FDG-PET imaging in different age groups.	137 (74) 20–80	Abdominal aorta, iliac, femoral, and popliteal arteries.	There was a significant difference in vascular uptake in FDG imaging between patients who were older than 60 years old (61%; 33/54) and those who were younger than 40 years old (34%; 12/35), with the *p*-value of 0.017. There was strong positive correlation between age groups and the prevalence of FDG vascular uptake (r = 0.99) as well.
Van der Valk et al. April 2016. Amsterdam, The Netherlands. [24]	Healthy subjects, patients with risk factors, and patients with CVD.	To compare the uptake of FDG in healthy subjects and patients with risk factors or with CVD.	83 (24) 61 ± 8	Carotid arteries and ascending aorta.	SUVmax gradually increased from healthy subjects to subjects with CVD. 96% of the patients with CVD risk factors and all patients with known CVD had at least one active slice on the imaging while only 48% of healthy subjects had the similar findings.
Tawakol et al. May 2006. Boston, Massachusetts. [25]	Patients with 70% to 99% carotid artery stenosis who were planned for CEA	To identify the correlation between FDG uptake and atherosclerotic plaque inflammation.	17 (6) 62 ± 6	Carotid arteries.	Macrophage staining and CD68 staining were used to assess the inflammation of atherosclerotic plaques, and TBR was used to assess FDG uptake. There was significant correlation between TBR and the macrophage staining (r = 0.70; *p* < 0.0001), as well as with the CD68 staining (r = 0.85; *p* < 0.0001).
Myers et al. Jan 2012. New York, USA. [26]	Patients with symptomatic femoral arterial disease.	To determine correlation between arterial FDG uptake and atherosclerotic plaque biomarkers.	30 67 ± 10	Aorta, carotid and femoral arteries.	There was no significant correlation between CD68 level which is the measure of macrophage content and TBR in the peripheral arteries (r = 0.21). The mean TBR of the carotid artery was 45% higher than that of peripheral artery (*p* < 0.05).
Yun et al. Jan 2002. PA, USA. [27]	Patients who were referred for various clinical evaluations.	To assess the FDG uptake in the different large arteries and the relationship with CVD risk factors.	156 (86) 0–96	Abdominal aorta, iliac, and proximal femoral arteries.	Age was the most significant risk factor in all three arteries studied. Hypercholesterolemia was another risk factor that had significant correlation FDG uptake in abdominal aorta and iliac arteries.
Bural et at. March 2008. PA, USA. [28]	Subjects who underwent FDG-PET imaging for the assessment of disease other than CVD.	To study how aging affects the changes of FDG uptake in large arteries.	149 (88) 5–83	Aorta, iliac and femoral arteries.	As patients aged, the mean SUVs of all arterial segments except abdominal aorta increased significantly (*p* < 0.01).
Strobl et al. Aug 2013. Munich, Germany. [29]	Subjects who underwent PET/CT scan for a noncardiovascular indication.	To evaluate the effect of age, gender and cardiovascular risk factors on vessel wall inflammation and calcified plaque burden.	315 (192) 57.8 ± 13.7	Thoracic and abdominal aorta, common carotid, and iliac arteries.	In all vessels studied, the inflammation of the vessel wall and the calcified plaque burden were significantly associated with age > 65 years (*p* < 0.05). However, the impact of other CVD risk factors differs depending on site.
Pasha et al. Feb 2015. PA, USA. [30]	Patients with melanoma.	To quantify FDG uptake in the aorta and peripheral arteries and evaluate the impact of age and CVD risk factors on the uptake of FDG.	76 (30) 22–91	Aorta, iliac, and femoral arteries.	Increasing age was significantly associated with increasing FDG uptake in the aorta and peripheral arteries. Nonetheless, the impact of cardiovascular risk factors on FDG uptake was only significant in the aorta (*p* < 0.05).
Rudd et al. April 2002. Cambridge, UK. [31]	Patients with symptomatic carotid atherosclerosis	To assess plaque inflammation in patients with symptomatic carotid artery disease using FDG-PET.	8 (2) 48–71	Carotid artery.	FDG-PET was able to visualize all symptomatic carotid plaques and no measurable uptake detected in normal carotid arteries. The accumulation rate of FDG was 27% higher in symptomatic lesions than in contralateral asymptomatic lesions.
Al-Zaghal et al. June 2020. PA, USA. [32]	Healthy controls and subjects with suspected lung malignancy.	To investigate the feasibility of FDG-PET/CT to detect pulmonary artery atherosclerosis and its correlation with abnormal PFT.	29 (0) 57–75	Pulmonary artery	Although the FDG uptake was higher in patients than in the control group, there was no statistically significant difference between non-COPD and COPD patients, indicating that the atherosclerotic process is a focal process.
Arani et al. April 2019. PA, USA. [33]	Healthy volunteers and patients with chest pain syndrome.	To study the association of FDG and NaF uptake with age and CVD risk factors.	123 (61) 48 ± 14	Abdominal aorta.	There was a positive correlation between NaF uptake with age (r = 0.35, *p* < 0.001) and 10 years FRS (r = 0.30, *p* < 0.001); however, no correlation was seen in the global uptake of FDG.
Blomberg et al. Oct 2016. Odense, Denmark. [34]	Healthy volunteers and patients with chest pain syndrome.	To identify the association between CVD risk with arterial inflammation, vascular calcification metabolism, and vascular calcium burden in a population at low CVD risk.	139 (67) 49 ± 14	Thoracic aorta.	Increased vascular calcification metabolism and vascular calcium burden were noted in subjects with unfavourable CVD risk profile. No association was noted with arterial inflammation.
Ben-Haim et al. Nov 2004. Haifa, Israel. [35]	Cancer patients who are 50 years or older.	To assess the imaging patterns of vascular-wall FDG uptake and CT calcifications in the wall of large arteries.	122 (47) 66 ± 9	Thoracic aorta, abdominal and carotid arteries.	Increased FDG uptake was present in 6% of sites (16% of patients) with concomitant vascular calcifications observed on CT and in 7% of sites (21% of patients) with no corresponding structural findings.
Tatsumi et al. Dec 2003. MD, USA. [36]	Patients who were known to have or were suspected of having cancers.	To evaluate the FDG uptake in the thoracic aortic wall by PET/CT imaging and compare the FDG uptake with the aortic wall calcification.	85 (39) 55 ± 16	Thoracic aorta and descending aorta	PET/CT depicted FDG uptake commonly in the thoracic aortic wall. The FDG uptake site was mostly distinct from the calcification site and may possibly be located in areas of metabolic activity of atherosclerotic changes.
Mierelles et al. Feb 2011. NY, USA. [37]	Patients with cancer.	To evaluate the stability of ^18^F-FDG uptake and vascular calcification using serial FDG scans.	100 (49) 20–80	Thoracic aorta.	Seventy percent of patients have positive ^18^F-FDG uptake on the first scan, however it was positive only in 55% of the patients on second scans. The co-existence of calcification and ^18^F-FDG uptake were only present in two cases.
Tawakol et al. Jan 2017. MA, USA. [38]	Individuals aged 30 years or older without known CVD or active cancer disorders.	To study the association of metabolic activity of amygdala with hematopoietic activity, arterial inflammation, and risk of future CVD.	293 (169) 45–65	Amygdala.	There was significant association between amygdalar activity with increased bone marrow activity, arterial inflammation, and risk of CVD events.
Blombery et al. June 2014. Odense, Denmark. [39]	Healthy controls and patients with chest pain.	To determine if delayed 18-FDG scans improves the evaluation of atherosclerotic plaque inflammation.	40 48.0 ± 14.9	Carotid arteries and thoracic aorta.	Delayed FDG imaging improves the evaluation, evidenced by significant positive relations observed between SCORE % and carotid and aortic SUVmax at 180 min but not at 90 min.

*n* = number; FDG = F-18 Fluorodeoxyglucose; PET = Positron Emission Tomography; CVD = cardiovascular disease; SUVmax = maximum standardized uptake value; TBR = target to background ratio (arterial wall SUVmax/venous background SUVmean); TBRmax = 90th percentile of the TBR; CEA = carotid artery endarterectomy; CAD = coronary artery disease; PAD = peripheral arterial disease; CPS = Calcified plaque score; wA-SUVmean = weighted-average mean standardized uptake value; ^18^F-NaF = ^18^F-sodium fluoride; FRS = Framingham risk score; MRI = magnetic resonance imaging; SCORE% = estimated 10-year risk for fatal cardiovascular disease; PFT = pulmonary function testing.

**Table 2:** Studies on the role of NaF in atherosclerosis.

Author Publication Year Country [Ref. #]	Subjects	Objectives	*n* (Female) Age in Years, Mean ± SD or Range	Arterial Segments	Findings
Joshi et al. Feb 2014. Edinburgh, UK. [14]	Patients with MI and stable angina.	To study the ability of NaF and FDG to identify ruptured and high-risk atherosclerotic plaques.	80 (7) 62 ± 8 (MI) 67 ± 8 (stable angina)	Proximal and mid-portions of the coronary arteries.	In 93% of the patients with MI, there was increased NaF uptake in the culprit plaque compared with non-culprit plaque (*p* < 0.0001) while there were no differences in coronary FDG uptake between culprit and nonculprit plaques.
Derlin et al. June 2010. Hamburg, Germany. [40]	Subjects who have undergone NaF PET/CT for the exclusion of bone metastases.	To study the relationship of vascular NaF uptake and arterial calcification in major arteries.	75 (48) 65.2 ± 12.3	Thoracic aorta, abdominal aorta, common carotid, iliac, and femoral arteries.	There was significant association between the vascular NaF uptake with the arterial calcification of the vessels studied (*p* < 0.0001).
Derlin et al. March 2011. Hamburg, Germany. [41]	Oncologic patients.	To correlate NaF accumulation in the common carotid arteries of neurologically asymptomatic patients with cardiovascular risk factors and carotid calcified plaque burden.	269 (166) 66.1 ± 12.4	Common carotid arteries.	There was significant association between NaF uptake with patients’ age (*p* < 0.0001), male (*p* < 0.0001), hypertension (*p* < 0.002), and hypercholesterolemia (*p* < 0.05). In conclusion, the correlation between the NaF uptake and number of present cardiovascular risk factors was strong (r = 0.30, *p* < 0.0001).
Behesti et al. August 2011. Linz, Austria. [42]	Patients who had undergone ^18^F-NaF-PET/CT for evaluation of malignancies.	To study the prevalence of regional (aorta) and global (cardiac) NaF uptake and the association with age.	51 (34) 29–90	Heart and aorta.	As patients aged, there was a significant increase in NaF uptake in the heart and aorta (*p* <0.01).
Piri et al. May 2021. Odense, Denmark. [43]	Healthy subjects and patients with angina pectoris.	To study the changes of carotid and aortic NaF uptake in 2 years.	49 (23) 21–75	Carotid arteries and aorta.	For both carotid arteries and aorta, patients with chest pain have slightly higher NaF uptake than the control group at baseline and after 2 years. However, the 2-year changes in both groups are very small and not significant.
Blomberg et al. Aug 2017. Odense, Denmark. [44]	Healthy subjects with low CVD risk.	To study the relationship between NaF uptake and CVD risk.	89 (42) 21–75	Coronary artery.	There were significant association between NaF uptake with female sex (*p* = 0.009), age (*p* = 0.002), and BMI (*p* < 0.001). The uptake of NaF increased linearly with the number of cardiovascular risk factors present (*p* < 0.001).
Janssen et al. Aug 2013. Hamburg, Germany. [45]	Oncologic patients.	To assess the correlation of NaF with cardiovascular risk factors and CPB.	409 (233) 25.1 ± 4.2	Femoral arteries.	As the number of CVD risk factors increased, the prevalence of NaF increased (*p* < 0.0001). There was a significant correlation between the NaF uptake with age, hypertension, hypercholesterolemia, diabetes, history of smoking, prior CVD, and CPB.
Zhang et al. April 2020. PA, USA. [46]	Healthy controls and subjects with suspected stable angina pectoris.	To assess the calcification of pulmonary arteries through NaF-PET/CT.	30 (6) 45 ± 8 (healthy controls) 56 ± 11 (at risk subjects)	Pulmonary arteries.	Patients at-risk demonstrated significantly higher NaF uptake compared to healthy controls (*p* < 0.05).
Kwiecinski et al. June 2020. CA, USA. [47]	Patients with known CAD.	To study the prediction of MI using NaF PET.	293 (46) 65 ± 9	Coronary artery.	There was an increase in NaF activity in 69% (203/293) of the patients and MI occurred only in these patients.
Kitagawa et al. Oct 2018. Hiroshima, Japan. [48]	Patients with ≥1 coronary atherosclerotic lesion detected on CCTA.	To investigate the utility of NaF uptake for predicting coronary events.	41 (8) 66 ± 9	Coronary artery.	Patients with coronary events had higher uptake than those without (*p* = 0.0034).
Patil et al. Aug 2020. PA, USA. [49]	Healthy, nondiabetic individuals.	To assess the correlation of TG/HDL ratio and subclinical coronary atherosclerosis.	68 (35) 41.7 ± 13.5	Coronary artery.	There was independent association between TG/HDL ratio and global cardiac aSUVmean (95% CI: 0.007–0.114, *p* = 0.027).
Rojulpote et al. Jun 2020. PA, USA. [50]	Healthy, non-dyslipidemic individuals.	To assess early atherosclerosis in individuals with a coronary calcium score of zero.	20 (8) 41.6 ± 13.8	Coronary artery.	Diastolic blood pressure and mean arterial pressure were correlated with cardiac NaF uptake independently.
Borja et al. Dec 2020. PA, USA. [51]	Individuals without known ASCVD.	To study the correlation of global coronary NaF quantification with ASCVD risk score.	61 (32) 53.4 ± 8.9	Coronary artery.	ASCVD risk score was significantly correlated to aSUVmean (r = 0.27, *p* = 0.03).
Gonuguntla et al. Sep 2020. PA, USA. [52]	Individuals with high risk factors of developing CVD events.	To evaluate the correlation of CHADS2 and CHA2DS2-VASc scores with NaF uptake in atherosclerotic plaque.	40 ( 22) 55 ± 11.9 SD	Coronary artery.	A higher CHADS2 and CHA2DS2-VASc scores correlate with a higher atherosclerotic burden, posting a greater risk of CVD events.
Dweck et al. April 2012. Cambridge, UK. [53]	Subjects with or without aortic valve disease.	To study the uptake of NaF as a marker of calcification and ^18^F-FDG as a marker of inflammation.	119 (38) 72 ± 8	Coronary arteries and aorta.	There was no increase in FDG uptake in both patients with atherosclerosis and control groups. However, higher rates of prior CVD events, angina, and higher FRS were noted in patients with increased coronary NaF activity.
Morbelli et al. Nov 2013. Genoa, Italy. [54]	Individuals with a history of breast or prostate cancer.	To investigate the relationship of the NaF uptake with FRS.	80 (60) 65.3 ± 8.2	Aorta, iliac, femoral, subclavian, and carotid arteries.	There was significant correlation between NaF uptake with all cardiovascular risk (age, diabetes, smoking, and systolic blood pressure), except the body mass index.
Li et al. June 2017. Vienna, Austria. [55]	Individuals with myeloma.	To investigate association between osteogenesis and inflammation during the progression of calcified plaque.	34 (8) 68 ± 9	Carotid arteries, aorta, and iliac arteries.	Noncalcified lesions have significant higher FDG uptakes than mildly or severely calcified lesions. During plaque progression, there was a concordant progression of inflammation and osteogenesis in 86% of noncalcified lesions, 81% of mildly calcified lesions, and less than 50% in severely calcified lesions.
Lee et al. Nov 2017. Seoul, Republic of Korea. [56]	Patients with suspected CAD.	To evaluate the NaF uptake in patients with CAD.	51 (6) 62.3 ± 8.2	Coronary artery.	The uptake of NaF in plaques with high-risk characteristics was significantly higher than in those without.
Marchesseau et al. Apr 2017. Singapore. [57]	Patients with STEMI undergoing primary PCI.	To study the combination of CT and NaF in detecting coronary lesions.	10 (1) 48 ± 7	Coronary artery.	NaF was able to detect myocardial scar tissues concurrently and its uptake was greater in high risk lesions than stable plaques.
Ishiwata et al. Aug 2017. Kanagawa, Japan. [58]	Patients with malignancy or orthopaedic disease.	To assess whether NaF PET/CT is able to predict progression of the CT calcium score.	34 (18) 57.5 ± 13.9	Aorta and common iliac artery.	There was a strong correlation between NaF uptake with calcium score progression, which was a predictor of future CVD risk, but no correlation was found between ^18^F-NaF uptake and calcification.
Fiz et al. Jan 2016. Genoa, Italy. [59]	Patients with breast or prostate cancer.	To study the correlation between thoracic and cardiac NaF uptake.	78 (44) 63.3 ± 8.2	Thoracic aorta.	Although there was correlation between TBR and CVR in the whole thoracic aorta (r = 0.67), the correlation was stronger in the descending thoracic segment (r = 0.75), compared to the aortic arch (r = 0.55) and the ascending segment (r = 0.53).
Arani et al. Nov 2020 PA, USA. [60]	Individuals with multiple myeloma and smoldering myeloma.	To assess the atherosclerosis risk in multiple myeloma and smoldering myeloma patients using NaF.	44 (14) 50–75	Aorta and whole heart.	Compared to controlled groups, patients with multiple myeloma demonstrated higher NaF uptake in the thoracic aorta and whole heart.
Takx et al. March 2020. Utrecht, The Netherlands. [61]	Subjects with type 2 diabetes and known arterial disease.	To evaluate the potential of NaF uptake as a determinant of arterial calcification in femoral arteries.	68 (16) 69 ± 8	Femoral arteries.	Higher NaF uptake was associated with higher CT calcium mass, total cholesterol, and HbA1c but not with smokers, male sex, or other medications.
Sorci et al. May 2020. Odense, Denmark. [62]	Healthy controls and patients who had experienced persistent chest pain.	To evaluate the benefit of utilizing NaF over calcium and FRS for potential preventive CAD intervention.	136 (68) 21–75	Coronary arteries.	In NaF PET/CT, patients have higher aSUVmeans compared to the control group, which is different from using the calcium score. Although FRS echoed the same, it was not sensitive enough to predict the patient status.
Piri et al. May 2021. Odense, Denmark. [63]	Healthy subjects with low CVD risk.	To evaluate the accuracy of CNN-based method for automated segmentation of the aortic wall in PET/CT scans.	49 (23) 52 ± 12	Aorta.	The automated CNN-based approach was faster than the manually obtained value and the SUV- mean values of both were comparable.
Piri et al. Aug 2021. Odense, Denmark. [64]	Healthy subjects and patients with chest pain.	To compare an AI- based method for cardiac segmentation in PET/CT scans with manual segmentation to assess global cardiac atherosclerosis burden.	49 (23) 52 ± 12	Heart.	The CNN-based method was faster and provided comparable values to the manually obtained value.

*n* = number; ^18^F-NaF = ^18^F-sodium fluoride; PET = Positron Emission Tomography; CPB = calcified plaque burden; MI = myocardial infarction; CAD = coronary artery disease; CCTA = coronary computed tomography angiography; TBRmax = maximum tissue: background ratio; TG = triglyceride; HDL = high-density lipoprotein; ASCVD = atherosclerotic cardiovascular disease; aSUVmean = average SUVmean; CVR = cardiovascular risk; CNN = convolutional neural networks; AI = artificial intelligence; CI = confidence interval; *p* = *p*-value; PCI = percutaneous coronary intervention.
